# Diagnostic accuracy of point-of-care lung ultrasound for community-acquired pneumonia in children in ambulatory settings: A systematic review and meta-analysis

**DOI:** 10.1177/1742271X241289726

**Published:** 2024-10-29

**Authors:** Helena Hughes-Davies, Umasha Ukwatte, Thomas R Fanshawe, Nia Roberts, Philip J Turner, Gail N Hayward, Chris Bird

**Affiliations:** 1 Nuffield Department of Primary Care Health Sciences, University of Oxford, Oxford, UK; 2 Birmingham Women’s and Children’s NHS Foundation Trust, Birmingham, UK

**Keywords:** Diagnostic ultrasound, children, pneumonia

## Abstract

**Introduction:**

To perform a systematic review of the diagnostic accuracy of point-of-care lung ultrasound, compared to chest radiography, in children and young people (0–21 years) who present to ambulatory settings with suspected community-acquired pneumonia.

**Methods:**

Registration: Prospero June 2021 CRD42021260552. Electronic searching performed on Medline, Embase, CINAHL and Science Citation Index from inception to 20 June 2023. Two researchers independently screened titles, abstracts, and full texts for study selection. Risk of bias was assessed using the Quality Assessment Tool for Diagnostic Accuracy Studies (QUADAS-2) tool. Meta-analysis of included studies.

**Results:**

The six studies included in this systematic review described point-of-care lung ultrasound performed primarily by paediatric emergency medicine clinicians on a total of 1099 paediatric patients, with a reference standard of chest radiography or chest radiography with clinical findings. The majority of included studies lacked clarity on training for the index test with potential bias around flow and timing of testing. Meta-analysis of the combined results of the included six studies calculated a pooled sensitivity of 90.9% (95% CI [85.5%, 94.4%]) and pooled specificity of 80.7% (95% CI [63.6%, 91.0%]).

**Conclusions:**

Point-of-care lung ultrasound has high sensitivity but lower specificity to diagnose acute pneumonia in children. Further research is needed which overcomes issues around training in point-of-care lung ultrasound, study design and reliability of the reference test (chest radiography) to better evidence the role of point-of-care lung ultrasound in diagnosing pneumonia in children in ambulatory and resource-limited settings.

## Introduction

The British Thoracic Society (BTS) advises bacterial pneumonia is a likely diagnosis in a child presenting with fever, chest recession and tachypnoea, although viruses and more rarely fungi are also aetiological agents.^1,2^ Pneumonia accounts for 14% of all deaths in children under the age of 5,^3^ with 90% of these deaths occurring in low-income countries.^4^ There are still around 2.5 million cases of paediatric pneumonia in high-income countries each year, of which 30%–50% result in hospitalisation.^1,5^

BTS guidelines recommend against the routine use of chest radiographs (CXR) or blood tests to diagnose pneumonia in children, with CXRs recommended only when the diagnosis is in doubt, or if the child is severely unwell.^6^ However, CXR is used in up to 73% of children with suspected pneumonia,^7,8^ potentially exposing them to unnecessary ionising radiation, despite being a cohort more radiosensitive when compared to adults.^9^ CXR is also time-intensive for clinicians, quality dependent on child behaviour and posture, with interpretation susceptible to considerable intra- and inter-observer variation.^8,10^

Lung ultrasound (LUS) at the point-of-care, or POC LUS, could offer an alternative diagnostic approach and potentially reduce the number of CXRs in children.^11^ It is effective in children who have a smaller lung volume and thoracic diameter, allowing easier visualisation of lung consolidation.^12^

LUS can be performed concurrently with a physical examination^13^ and can be taught relatively quickly to clinicians without ultrasound experience.^14^ The International Liaison Committee on Lung Ultrasound considers LUS a basic sonographic technique^15^ which clinicians can interpret accurately after 25 scans.^16^ Point-of-care ultrasound is portable or handheld and can be done at the bedside by the attending physician, which allows for immediate interpretation and management, which would also be ideal for community and resource-limited settings.

Despite evidence supporting the use of POC LUS,^17–19^ no current guidelines recommend its use. The aim of this study was to perform a systematic review of the available evidence for the diagnostic accuracy of point-of-care LUS when performed by a clinician in an acute setting, compared to a standard CXR for pneumonia in children.

## Methods

Reporting of this systematic review follows the diagnostic test accuracy extension of the Preferred Reporting Items for Systematic Reviews and Meta-Analyses (PRISMA-DTA) statement,^20^ with the PRISMA checklist submitted with the manuscript. The review was registered with Prospero June 2021, number CRD42021260552.

### Eligibility criteria and definitions

Studies were considered for inclusion if the study population included children and young adults between the ages of 0 and 21 (accounting for variation in age cut-offs of paediatric services globally) presenting with suspected pneumonia to ambulatory care settings including primary care clinics, hospital outpatient clinics, emergency departments, walk-in centres and acute clinics in resource-limited regions. If studies included adults and children, only studies that distinguished quantitative outcomes for children alone were considered.

The index test was any POC LUS used with real-time analysis to detect lung consolidation. The ultrasound could be conducted by any frontline clinician managing sick children who was not exclusively a professional sonographer or radiographer. We originally defined sufficient experience as a minimum of 25 scans, as recommended by the American College of Emergency Physicians^16^ but found this made the inclusion criteria too narrow. The adjusted inclusion criteria considered studies that described clinician training had taken place, even in cases where the number of scans attempted before the study was not disclosed or the recommended 25 scan benchmark was not met. The reference standard considered was a CXR or CXR in combination with clinical findings. Studies that did not report individual patient-level diagnostic results were excluded.

### Search strategy

Medline, Embase, CINAHL and Science Citation Index were searched from inception to 20 June 2023 with no language restrictions. The search, which also included ultrasound for diagnoses other than pneumonia, included the following terms: (children OR adolescent OR infant) AND (ultrasonography) AND (point-of-care OR hand-held OR portable). The full search strategy can be seen in Supplemental Online Material 1. From this broader search we identified studies that met our inclusion criteria for this study focused on LUS for pneumonia.

### Study selection

We included retrospective and prospective cohort studies meeting our inclusion criteria. Two independent reviewers identified the relevant studies by reading titles and abstracts (H.H.-D., C.B.). Disagreements were arbitrated by a third reviewer (U.U.). Full text for the studies was retrieved when both reviewers agreed a study was relevant or if there was insufficient information in the abstract to form a judgement. The full text of the studies was reviewed to judge eligibility.

### Data extraction and quality assessment

One reviewer extracted data from the selected studies using a data extraction form (H.H.-D.), which was then checked by a second reviewer (U.U.), with any disagreements adjudicated by a third reviewer (C.B.). Extraction included study characteristics, participants, index and reference test and results. Contact was attempted with authors whose studies did not provide all the required details. Both reviewers used the QUADAS-2 tool^21,22^ to assess the quality of studies. Disagreements were decided by the third, independent reviewer.

### Data analysis

Data for diagnostic accuracy was summarised in a 2 × 2 classification table, where LUS was the index test and CXR the reference test, to categorise patients with or without pneumonia. A random effects bivariate meta-analysis model^23^ was fitted using the R package ‘mada’ to calculate pooled sensitivities and specificities, with a continuity correction of 0.5 applied to studies with zero counts.^24^ The primary analysis used all studies. We planned to perform a subgroup analysis using only studies that reached the threshold for sufficient clinician experience, but this was not possible because too few studies were found that met this threshold.

### Patient and public involvement (PPI)

The planned systematic review was presented to the NIHR Community Healthcare MIC, Oxford’s PPI group for acute paediatrics in 2021 who deemed the research was relevant to diagnostics used in the delivery of acute child health.

## Results

### Study characteristics and definitions

**Figure 1.**
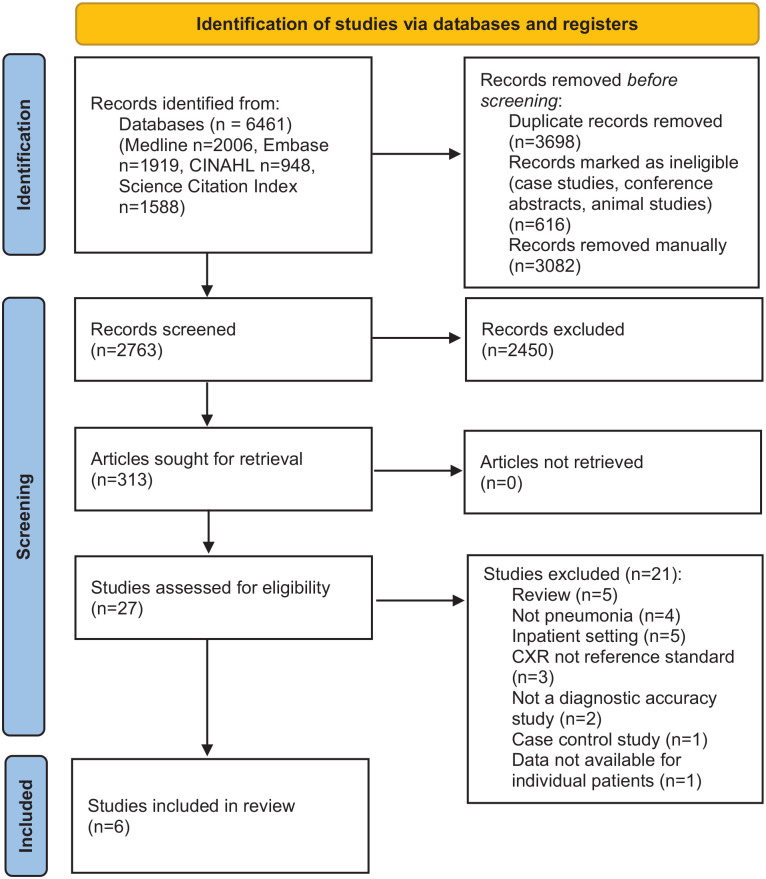
PRISMA 2020 flow diagram for diagnostic accuracy of point-of-care lung ultrasound for community-acquired pneumonia in children in ambulatory settings: a systematic review and meta-analysis.

The search identified 6461 studies, of which 3698 were excluded due to being duplicates, case reports, conference abstracts or animal studies (Medline – 2006, Embase – 1919, CINAHL – 948, Science citation index – 1588). Of the 2763 studies then screened, six were eligible for inclusion in the systematic review^8,25–29^ (see PRISMA flow diagram, Figure 1).^30^

Table 1 provides a summary of study characteristics. All were prospective observational studies (total sample size 1099 patients). Five of the six studies were conducted in children’s EDs,^8,25,26,28,29^ one of which was in a resource-limited setting,^29^ while the remaining study took place in primary care.^27^ Participant recruitment methods included convenience sampling^8,26,28^ and consecutive sampling,^27^ although sampling methods for two studies were unclear.^25,29^ The average age (reported as either mean or median) in the included studies ranged from 1.3 to 8.9 years. The percentage of male participants was close to 50% in all studies, where reported, and the prevalence of pneumonia ranged from 18% to 92%.

**Table 1.** Summary study characteristics.

	Setting	Country	No. of participants	Age Range	Reference standard	Reference standard operator	Inclusion criteria	Exclusion criteria	Experience of clinicians performing scan	Diagnostic criteria
Shah et al.^8^	ED	USA	200	0–21 yrs	CXR	Radiologist	Suspicion of CAP.	Patients who arrived in the ED with a chest radiograph, patients with previously confirmed diagnosis of pneumonia, patients who had hemodynamic instability.	Sonologist with 1 hour of ultrasound training.	Lung consolidation with sonographic air bronchograms.
Guerra et al.^25^	ED	Italy	222	3 months–16 yrs	CXR	Radiologist	Temperature > 38.5°C, moderate to severe respiratory distress, moderate to severe breathlessness, moderate to severe retractions, nasal flaring, grunting respiration, oxygen saturation < 92% on room air.	Children with respiratory distress due to asthma or with clinical diagnosis of mild/moderate bronchiolitis.	Paediatricians with specific LUS expertise, who had attended a LUS course and had supervised practical training	Lung consolidation with sonographic air bronchograms
Lissaman et al.^26^	ED	Australia	97	1 month–18 yrs	CXR	Radiologist	Children who had received a CXR for possible pneumonia.	Children with prior CXR for the same illness or requiring life support.	Paediatric emergency medicine fellow and final-year medical student. Both trained to perform LUS with a 1 hour online video and 1 hour practical tutorial.	LUS was interpreted by two sonologists
Rodriguez-Contreras et al.^27^	Primary care centre	Spain	15	5–14 yrs (paediatric cohort)	CXR	Radiologist	Children and adults with clinically suspected CAP.	Hospital admission within past 30 days, pneumonia already diagnosed, children with previous diagnosis of viral wheeze.	Clinicians had 40 hours training in abdominal ultrasound and 5 hours of training for LUS.	LUS was considered positive if any of the following were present: 1 or more consolidations, greater than 1 cm or focal, unilateral, or asymmetrical bilateral B-lines pattern.
Samson et al.^28^	ED	Spain	200	0–15 yrs	CXR	Radiologist	Patients with clinical suspicions of CAP who required CXR for evaluation.	Patients who already a CXR, haemodynamically instable, clinical bronchiolitis, patients who had already received antibiotics. Children with chronic chest diseases.	Paediatricians and paediatrician residents had 2-hour lectures and a 1-hour hands-on training session.	Classified as pneumonia if lung consolidation or sonographic air bronchograms.
Amatya et al.^29^	ED	Nepal	365	0–5 years	CXR	Radiologist	Patients from with clinical suspicion of CAP who had a CXR performed	Children with no CXR	Clinicians had varied levels of ultrasound training.	Classified as pneumonia if unilateral focal B-lines or subpleural consolidation

ED: emergency department; yrs: years of age; CXR: chest radiograph; LUS: lung ultrasound; CAP: community acquired pneumonia.

Training for frontline clinicians varied from an hour-long programme (consisting of a lecture and practical session) to 5 hours of training, although two studies provided insufficient detail around LUS training and experience.^25,29^ Ultrasound probe type and frequency varied between studies and included the use of a curvilinear probe,^29^ convex probe^25^ or a linear probe with 3.5–5,^25^ 7.5–10,^8,25^ 8–14^27^ and 6–15 MHz range of frequencies reported.^28^ Two of the included studies were unclear about the range of the frequency of the transducer^26,29^ (see Table 4). All studies used real-time evaluation and defined lung consolidation (an area where air is replaced by fluid and presses on the pleural surface) as a sign of pneumonia on CXR. For all studies, the reference test was CXR interpreted by either a radiologist or a patient-facing clinician.

### Study quality

Table 2 summarises the QUADAS-2 quality assessment (full review can be found in Supplemental Online Material 3). One study excluded children under 5 years of age, a cohort who commonly present with pneumonia, meaning applicability concerns were high for patient selection.^27^ Five of the six included studies did not meet our review’s original 25-scan experience threshold for inclusion (results for diagnostic accuracy are separated out by experience in Table 3).^8,25,26,28,29^ Rodriguez-Contreras et al.’s study was the only one at low risk of bias for the index test as it detailed sufficient experience with scanning (> 25 scans).^27^

**Table 2.** Summary of quality assessment using the QUADAS-2 tool.

	Risk of bias				Applicability Concerns		
	Patient selection	Index test	Reference standard	Flow and timing	Patient selection	Index test	Reference standard
Shah et al.^8^	Low	Unclear	Low	Unclear	Low	Low	Low
Guerra et al.^25^	Low	Unclear	Low	Unclear	Low	Unclear	Low
Lissaman et al.^26^	Unclear	Unclear	Low	Low	Low	Low	Low
Rodriguez-Contreras et al.^27^	Unclear	Low	Low	Low	High	Low	Low
Samson et al.^28^	Low	Unclear	Low	Unclear	Low	Low	Low
Amatya et al.^29^	Low	Unclear	Low	Unclear	Low	Low	Low

**Table 3.** Diagnostic accuracy results from included studies, with data presented to differentiate between clinicians experienced in POC LUS and inexperienced clinicians.

Study	TP	FP	FN	TN	Estimated sensitivity [95% CI]	Estimated specificity [95% CI]	Estimated prevalence (%)
*Clinicians experienced with POC LUS (>* *25 scans)*							
Rodriguez-Contreras et al.^27^	8	1	0	6	100.0 [67.6, 100.0]	85.7 [48.7, 97.4]	53.3
*Clinicians inexperienced with POC LUS (<* *25 scans or not stated)*							
Guerra et al.^*25^	197	10	7	8	96.6 [93.1, 98.3]	44.4 [24.6, 66.3]	91.9
Lissaman et al.^26^	40	17	4	36	90.9 [78.8, 96.4]	67.9 [54.5, 78.9]	45.4
Samson et al.^28^	74	6	11	109	87.1 [78.3, 92.6]	94.8 [89.1, 97.6]	42.5
Amatya et al.^29^	75	39	9	242	89.3 [80.9, 94.3]	86.1 [81.6, 89.7]	23.0
Shah et al.^8^	31	18	5	146	86.1 [71.3, 93.9]	89.0 [83.3, 92.9]	18.0

TP: true positive; FP: false positive; FN: false negative; TN: true negative; CI: confidence interval; POC: point-of-care; LUS: lung ultrasound.

* The total number of TP for Guerra et al.^25^ includes 7 LUS-positive cases that were initially negative on CXR but were subsequently found to be positive on CXR review.

All six studies had low risk of bias and low applicability concerns for conduct and interpretation of the reference standard as they ensured the clinicians interpreting the CXR were blinded to the results of the LUS. However, four of the six studies had unclear risk of bias for flow and timing as they did not specify time between performing ultrasonography and chest radiography.^8,25,28,29^

### Diagnostic accuracy

**Figure 2.**
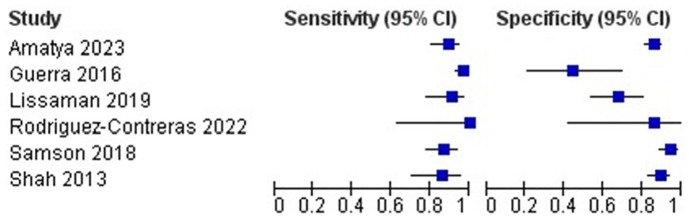
Forest plot of included studies.

Table 3 and Figure 2 (forest plot) detail the diagnostic accuracy estimates for the six included studies. All studies had an estimated sensitivity above 86%. The range was from 86.1% to 100%, although the highest sensitivity came from a study including only 15 children.^27^ Specificity varied more than sensitivity, with a range from 44.4% to 94.8%. Prevalence of pneumonia in the included patients also varied from 18% to 92%.

### Meta-analysis

We pooled all six studies that met our inclusion criteria. The pooled sensitivity was 90.9% (95% CI [85.5%, 94.4%]) and the pooled specificity was 80.7% (95% CI [63.6%, 91.0%]). As a meta-analysis of diagnostic accuracy studies, there is no widely used measure of heterogeneity, although this study found high heterogeneity in the pooled specificity estimate while we also observed considerable methodological heterogeneity in the different study designs.

## Discussion

### Summary of results

In six studies compromising 1099 children with suspected pneumonia attending EDs (one in a resource-limited setting) and one primary care centre, LUS had high sensitivity but lower specificity for diagnosing pneumonia as identified by CXR. The high pooled sensitivity (90.9%) suggests POC LUS, even with the small amount of training provided to clinicians involved in these studies, could be an effective tool for frontline clinicians to identify community-acquired pneumonia (CAP) in children instead of CXR. However, our pooled specificity is lower (80.7%). Depending on how often a CXR result is believed by clinicians to confirm the absence of bacterial pneumonia^31^ low specificity could lead to more children receiving unnecessary antibiotics.

### Strengths and limitations

#### Strengths

This prospectively registered review was conducted following best practice with a thorough search of the literature. The study protocol screened for studies in ambulatory, acute settings, with POC LUS carried out by non-radiologists/sonographers, whereas previous studies have focused on inpatient and intensive care settings, often with radiologist/sonologist operators, making this study more relevant to a wider patient population, particularly for resource-limited settings.

#### Limitations

The main limitation of this review is the quality and number of studies available on this topic. Only six studies met our inclusion criteria. Only one of the included studies evaluated the use of POC LUS in a resource-limited setting^29^ where the need is greatest and only one small study was set in primary care, where imaging could help avoid onward referrals.^27^ Our results are best interpreted in the context of a well-resourced children’s ED.

The pooled specificity was lower than the pooled sensitivity, with heterogeneity between studies. One explanation could be the limitations of the CXR as a reference standard. CXRs cannot identify small consolidations, which may have resulted in POC LUS identifying smaller consolidations which were subsequently classified as false positives when compared to CXR.^7^ When Shah et al.^8^ performed a subgroup analysis excluding lung consolidations less than 1 cm in diameter, sensitivity remained the same at 86% but specificity increased from 89% to 97%. Furthermore, while traditionally epidemiology and diagnostic accuracy studies for CAP have used CXR as the reference standard, many national guidelines no longer advocate CXRs routinely in the diagnosis of CAP, relying more on clinical findings.^1^ Reali et al.^32^ combined CXR with clinical findings as the reference standard for patients already admitted to hospital. This study did not meet our inclusion criteria as it included patients admitted to hospital. It found a specificity of 96%, higher than our pooled specificity, but admitted patients would be more likely to have positive findings on CXR.

The studies included in this review used varying ultrasound devices with different probe types and associated frequencies, further contributing to heterogeneity between studies. The range of frequencies determines the depth of penetration as well as image quality (with low frequencies penetrating deeper with the caveat of lower image quality), while the shape (curvilinear or linear) can determine the shape of image captured (see Table 4).^33^

**Table 4.** Ultrasound device used, transducer frequency and probe type.

Study	Ultrasound brand	Frequency	Transducer/probe
Shah et al.^8^	Micromaxx; Sonosite GS60; Siemens	7.5–10 MHz	Linear array transducer
Samson et al.^28^	S-Nerve Sonosite	6–15 MHz	Linear probe
Lissaman et al.^26^	Zonare	Unclear	Linear probe
Guerra et al.^25^	MyLab 25	7.5–10 MHz 3.5–5 MHz	Linear probe Convex probe
Amatya et al.^29^	Sonosite M Turbo	Unclear	Curvilinear probe
Rodriguez-Contreras et al.^27^	MyLabSix MyLab 40 DC-N3 Logiq F6 Logiq C5 Premium MicroMaxx	8–14 MHz	Linear probe

MHz: megahertz.

### Comparison with the literature

Our pooled sensitivity is supported by recent meta-analyses of POC LUS which report sensitivities of 94%^17^ and 96%^18^ in paediatric populations. Compared to our results, these reviews reported slightly higher specificities of 93% and 90%, respectively, although some of these studies used LUS performed by radiographers rather than POC LUS performed by front line clinicians and included hospitalised, neonatal and critically ill patients, who have a higher likelihood of positive findings for CAP on both CXR and POC LUS.

### Implications for research and practice

Our results demonstrate a potential role for LUS to diagnose CAP in children in ambulatory and resource-limited settings. While CXR remains the current reference standard in most clinical settings, it has limited sensitivity and specificity. Ultrasound devices offer greater agility as they can be deployed at the bedside, avoid risks associated with ionising radiation, which are notably higher for paediatric populations, and are an inexpensive diagnostic modality when compared to CXR.^29,34^ However, our lower pooled specificity means further study is required in populations of patients where POC LUS is likely to add most value. Further investigation is needed before use in children with acute respiratory infections, to ensure robust evaluation of POC LUS’ effect on antibiotic prescribing (e.g. POC LUS’ greater sensitivity might lead to unnecessary antibiotic use for viral infections) and hospital referral/admission.

Particular attention in new studies should be given to defining appropriate training in LUS for clinicians and defining a reference standard more closely representative of clinical practice in primary care and resource-limited settings. In these contexts where there may be greater emphasis on clinical findings alongside CXR, recruiting an appropriate sample size to detect a meaningful effect, and using inclusion criteria that better reflect undifferentiated acute respiratory illness in children will provide research findings that are more able to be practically applied.

Studies of children presenting to primary care and resource-limited settings could lead to wider adoption of POC LUS outside well-resourced hospital settings.

## Conclusion

POC LUS for diagnosing paediatric pneumonia has a high pooled sensitivity but lower specificity in ambulatory settings, including a resource-limited setting. Better designed studies are needed to understand performance and likely impact of this new technology across frontline paediatric settings, particularly on antibiotic stewardship.

## Supplemental Material

sj-docx-1-ult-10.1177_1742271X241289726 – Supplemental material for Diagnostic accuracy of point-of-care lung ultrasound for community-acquired pneumonia in children in ambulatory settings: A systematic review and meta-analysis Supplemental material, sj-docx-1-ult-10.1177_1742271X241289726 for Diagnostic accuracy of point-of-care lung ultrasound for community-acquired pneumonia in children in ambulatory settings: A systematic review and meta-analysis by Helena Hughes-Davies, Umasha Ukwatte, Thomas R Fanshawe, Nia Roberts, Philip J Turner, Gail N Hayward and Chris Bird in Ultrasound

sj-docx-2-ult-10.1177_1742271X241289726 – Supplemental material for Diagnostic accuracy of point-of-care lung ultrasound for community-acquired pneumonia in children in ambulatory settings: A systematic review and meta-analysis Supplemental material, sj-docx-2-ult-10.1177_1742271X241289726 for Diagnostic accuracy of point-of-care lung ultrasound for community-acquired pneumonia in children in ambulatory settings: A systematic review and meta-analysis by Helena Hughes-Davies, Umasha Ukwatte, Thomas R Fanshawe, Nia Roberts, Philip J Turner, Gail N Hayward and Chris Bird in Ultrasound

## References

[1] Harris M Clark J Coote N , et al. British Thoracic Society guidelines for the management of community acquired pneumonia in children: update 2011. Thorax 2011; 66: ii1–ii23. 10.1136/thoraxjnl-2011-200598 21903691

[2] Principi N Esposito S . Management of severe community-acquired pneumonia of children in developing and developed countries. Thorax 2011; 66: 815–822. 20965930 10.1136/thx.2010.142604

[3] World Health Organization (WHO). Pneumonia in children, https://www.who.int/news-room/fact-sheets/detail/pneumonia (2022, accessed 21 August 2024).

[4] Chavez MA Naithani N Gilman RH , et al. Agreement between the World Health Organization algorithm and lung consolidation identified using point-of-care ultrasound for the diagnosis of childhood pneumonia by general practitioners. Lung 2015; 193: 531–538. 25921013 10.1007/s00408-015-9730-x

[5] Murdoch D Howie SRC . Global childhood pneumonia: the good news, the bad news, and the way ahead. Lancet Glob Health 2019; 7: e4–e5. 30497987 10.1016/S2214-109X(18)30446-7

[6] British Thoracic Society. Quality improvement tool – paediatric community acquired pneumonia, 2019, https://www.brit-thoracic.org.uk/quality-improvement/clinical-resources/paediatric-community-acquired-pneumonia/ (2019, accessed 22 August 2024).

[7] Florin T French B Zorc J , et al. Variation in emergency department diagnostic testing and disposition outcomes in pneumonia. Pediatrics 2013; 132: 237–244. 23878049 10.1542/peds.2013-0179

[8] Shah VP Tunik MG Tsung JW . Prospective evaluation of point-of-care ultrasonography for the diagnosis of pneumonia in children and young adults. JAMA Pediatr 2013; 167: 119–125. 23229753 10.1001/2013.jamapediatrics.107

[9] European Union. Radiation protection 118: referral guidelines for imaging, https://rcc-uk.org/wp-content/uploads/2013/12/Radiation-Protection-118-%E2%80%93-Referral-Guidelines-for-Imaging.pdf (2000, accessed 22 August 2024).

[10] Elemraid MA Muller M Spencer DA , et al. Accuracy of the interpretation of chest radiographs for the diagnosis of paediatric pneumonia. PLoS One 2014; 9: e106051. 25148361 10.1371/journal.pone.0106051 PMC4141860

[11] Pardue Jones B Tay ET Elikashvili I , et al. Feasibility and safety of substituting lung ultrasonography for chest radiography when diagnosing pneumonia in children: a randomized controlled trial. Chest 2016; 150: 131–138. 26923626 10.1016/j.chest.2016.02.643

[12] Biagi C Pierantoni L Baldazzi M , et al. Lung ultrasound for the diagnosis of pneumonia in children with acute bronchiolitis. BMC Pulm Med 2018; 18: 191. 30526548 10.1186/s12890-018-0750-1 PMC6286612

[13] Sherman J Abo A . Evaluation of pulmonary emergencies using point-of-care ultrasound in the pediatric emergency department: a review. Clin Pediatr Emerg Med 2015; 16: 244–254.

[14] House DR Amatya Y Nti B , et al. Lung ultrasound training and evaluation for proficiency among physicians in a low-resource setting. Ultrasound J 2021; 13: 34. 34191145 10.1186/s13089-021-00236-4 PMC8245620

[15] Volpicelli G Elbarbary M Blaivas M , et al. International evidence-based recommendations for point-of-care lung ultrasound. Intensive Care Med 2012; 38: 577–591. 22392031 10.1007/s00134-012-2513-4

[16] American College of Emergency Physicians. Ultrasound guidelines: emergency, point-of-care and clinical ultrasound guidelines in medicine. Ann Emerg Med 2017; 69: e27–e54. 28442101 10.1016/j.annemergmed.2016.08.457

[17] Pereda MA Chavez MA Hooper-Miele CC , et al. Lung ultrasound for the diagnosis of pneumonia in children: a meta-analysis. Pediatrics 2015; 135: 714–722. 25780071 10.1542/peds.2014-2833 PMC9923609

[18] Tsou P Chen KP Wang Y , et al. Diagnostic accuracy of lung ultrasound performed by novice versus advanced sonographers for pneumonia in children: a systematic review and meta-analysis. Acad Emerg Med 2019; 26: 1074–1088. 31211896 10.1111/acem.13818

[19] Orso D Ban A Guglielmo N . Lung ultrasound in diagnosing pneumonia in childhood: a systematic review and meta-analysis. J Ultrasound 2018; 21: 183–195. 29931473 10.1007/s40477-018-0306-5 PMC6113181

[20] Page MJ McKenzie JE Bossuyt PM , et al. The PRISMA 2020 statement: an updated guideline for reporting systematic reviews. BMJ 2021; 372: n71. 33782057 10.1136/bmj.n71 PMC8005924

[21] University of Bristol. QUADAS-2, https://www.bristol.ac.uk/population-health-sciences/projects/quadas/quadas-2/ (2021, accessed 22 August 2024).

[22] Whiting PF Rutjes AW Westwood ME , et al. QUADAS-2: a revised tool for the quality assessment of diagnostic accuracy studies. Ann Intern Med 2011; 155: 529–536. 22007046 10.7326/0003-4819-155-8-201110180-00009

[23] Chu H Cole SR . Bivariate meta-analysis of sensitivity and specificity with sparse data: a generalized linear mixed model approach. J Clin Epidemiol 2006; 59: 1331–1332; author reply 1332–1333. 17098577 10.1016/j.jclinepi.2006.06.011

[24] Doebler P Holling H Sousa-Pinto B . Meta-analysis of diagnostic accuracy with mada, https://cran.r-project.org/web/packages/mada/vignettes/mada.pdf (2022, accessed 22 August 2024).

[25] Guerra M Crichiutti G Pecile P , et al. Ultrasound detection of pneumonia in febrile children with respiratory distress: a prospective study. Eur J Pediatr 2016; 175: 163–170. 26283293 10.1007/s00431-015-2611-8

[26] Lissaman C Kanjanauptom P Ong C , et al. Prospective observational study of point-of-care ultrasound for diagnosing pneumonia. Arch Dis Child 2019; 104: 12–18. 29880545 10.1136/archdischild-2017-314496

[27] Rodriguez-Contreras F Calvo-Cebrian A Diaz-Lazaro J , et al. Lung ultrasound performed by primary care physicians for clinically suspected community-acquired pneumonia: a multicenter prospective study. Ann Fam Med 2022; 20: 227–236. 35606120 10.1370/afm.2796 PMC9199040

[28] Samson F Gorostiza I Gonzalez A , et al. Prospective evaluation of clinical lung ultrasonography in the diagnosis of community-acquired pneumonia in a pediatric emergency department. Eur J Emerg Med 2018; 25: 65–70. 27536810 10.1097/MEJ.0000000000000418

[29] Amatya Y Russell FM Rijal S , et al. Bedside lung ultrasound for the diagnosis of pneumonia in children presenting to an emergency department in a resource-limited setting. Int J Emerg Med 2023; 16: 2. 36624366 10.1186/s12245-022-00474-w PMC9828356

[30] Haddaway NR Page MJ Pritchard CC , et al. *PRISMA2020*: an R package and Shiny app for producing PRISMA 2020-compliant flow diagrams, with interactivity for optimised digital transparency and Open Synthesis. Campbell Syst Rev 2022; 18: e1230. 10.1002/cl2.1230 PMC8958186 36911350

[31] Lipshaw MJ Florin TA Krueger S , et al. Factors associated with antibiotic prescribing and outcomes for pediatric pneumonia in the emergency department. Pediatr Emerg Care 2021; 37: e1033–e1038. 31290801 10.1097/PEC.0000000000001892 PMC6946906

[32] Reali F Sferrazza Papa GF Carlucci P , et al. Can lung ultrasound replace chest radiography for the diagnosis of pneumonia in hospitalized children? Respiration 2014; 88(2): 112–115. 24992951 10.1159/000362692

[33] Szabo TL Lewin PA . Ultrasound transducer selection in clinical imaging practice. J Ultrasound Med 2013; 32: 573–582. 23525382 10.7863/jum.2013.32.4.573

[34] O’Grady KF Torzillo PJ Frawley K , et al. The radiological diagnosis of pneumonia in children. Pneumonia 2014; 5: 38–51. 31641573 10.15172/pneu.2014.5/482 PMC5922330

