# Supplementary material for: Diagnostic accuracy of point-of-care lung ultrasound for community-acquired pneumonia in children in ambulatory settings: A systematic review and meta-analysis
Source: Ultrasound. 2024 Oct 29;33(3):197–205. doi: 10.1177/1742271X241289726 (PMC11563508; doi:10.1177/1742271X241289726)
Supplement: sj-docx-1-ult-10.1177_1742271X241289726 – Supplemental material for Diagnostic accuracy of point-of-care lung ultrasound for community-acquired pneumonia in children in ambulatory settings: A systematic review and meta-analysis [file sj-docx-1-ult-10.1177_1742271X241289726.docx]

SUPPLEMENTARY ONLINE MATERIAL

**Supplementary Online Material 1** – *Search Strategy for MEDLINE*

| 1 | adolescent/ or exp child/ or exp infant/ |
| --- | --- |
| 2 | (child* or infan* or baby or babies or toddler? or schoolchild* or preschool* or pre-school* or p?ediatric* or girl? or boy? or adolescen* or teen*).ti,ab. |
| 3 | 1 or 2 |
| 4 | Exp Ultrasonography/ |
| 5 | (ultrasound* or ultra-sound or ultrasonogra* or ultra-sonogra* or sonogra*).ti. |
| 6 | 4 or 5 |
| 7 | Exp Point-of-Care Systems/ |
| 8 | (poc or poct or “point of care” or handheld or hand-held or portable or focused).ti. |
| 9 | 7 and 8 |
| 10 | 6 and 9 |
| 11 | ((ppoc or poct “point of care” or handheld or hand-held or portable or focused) adj5 (ultrasound* or ultra-sound or ultrasonogra* or ultra-sonogra* or sonogra*)).ti,ab. |
| 12 | pocus.ti,ab |
| 13 | 10 or 11 or 12 |
| 14 | 3 and 13 |
| 15 | (comment or editorial or letter or news or “review”).pt. or case report.ti,ab |
| 16 | 14 not 15 |
| 17 | Limit 14 to (“systematic review” or “reviews (maximizes specificity)”) |
| 18 | 16 or 17 |
| 19 | (exp animals/ or nonhuman/) not human/ |
| 20 | 18 not 21 |
| 21 | (2023* or 2022*). yr, ed, ez |
| 22 | 20 and 21 |
