# Supplementary material for: Diagnostic accuracy of point-of-care lung ultrasound for community-acquired pneumonia in children in ambulatory settings: A systematic review and meta-analysis
Source: Ultrasound. 2024 Oct 29;33(3):197–205. doi: 10.1177/1742271X241289726 (PMC11563508; doi:10.1177/1742271X241289726)
Supplement: sj-docx-2-ult-10.1177_1742271X241289726 – Supplemental material for Diagnostic accuracy of point-of-care lung ultrasound for community-acquired pneumonia in children in ambulatory settings: A systematic review and meta-analysis [file sj-docx-2-ult-10.1177_1742271X241289726.docx]

SUPPLEMENTARY ONLINE MATERIAL

**Supplementary Online Material 2** – *expanded QUADAS review*

Patient selection

|  | **Was a consecutive or random sample of patients enrolled?** | **Was a case-control design avoided?** | **Did the study avoid inappropriate exclusion?** | **What is the risk that the selection of patients has introduced bias?** | **What concern do you have that the included patients do not match the review question?** |
| --- | --- | --- | --- | --- | --- |
| Shah (2013) | No | Yes | Yes | Low | Low |
| Guerra (2016) | Unclear | Yes | Yes | Low | Low |
| Lissaman (2019) | No | Yes | Yes | Unclear | Low |
| Rodriguez-Contrera (2022) | Yes | Yes | No | Unclear | High |
| Samson (2018) | No | Yes | Yes | Low | Low |
| Amatya (2023) | No | Yes | Yes | Low | Low |

Index test

|  | **Were the results of the index test interpreted without knowledge of the results of the reference standard?** | **Had test operators had appropriate training?** | **Did the study provide a clear definition of what was considered a “positive” result?** | **If a threshold was used, was it pre-specified?** | **Could the conduct or interpretation of the index test have introduced bias?** | **Is there a concern that the index test, its conduct, or its interpretation differs from the review question?** |
| --- | --- | --- | --- | --- | --- | --- |
| Shah (2013) | Yes | Unclear | Yes | Yes | Unclear | Low |
| Guerra (2016) | Yes | Unclear | Yes | Yes | Unclear | Unclear |
| Lissaman (2019) | Yes | Unclear | Yes | Yes | Unclear | Low |
| Rodriguez-Contrera (2022) | Yes | Yes | Yes | Yes | Low | Low |
| Samson (2018) | Yes | Unclear | Yes | Yes | Unclear | Low |
| Amatya (2023) | Yes | Unclear | Yes | Yes | Unclear | Low |

Reference standard

|  | **Describe the reference standard, how it was conducted, and how it was interpreted** | **Is the reference standard likely to correctly classify the target condition?** | **Were the reference standard results interpreted without the knowledge of the results of the index test?** | **Could the reference standard, its conduct or its interpretation have introduced bias?** | **Is there concern that the target condition as defined by the reference standard does not match the review question?** |
| --- | --- | --- | --- | --- | --- |
| Shah (2013) | CXR analysed by the attending pediatric radiologist | Yes | Yes | Low | Low |
| Guerra (2016) | CXR analysed by the radiologist on duty, informed of the clincial conditions but unaware of LUS findings | Yes | Yes | Low | Low |
| Lissaman (2019) | Chest radiograph performed and interpreted by a clinician | Yes | Yes | Low | Low |
| Rodriguez-Contreras (2022) | CXR interpreted by a radiologist on duty | Yes | Yes | Low | Low |
| Samson (2018) | CXR analysed by 2 radiologists + consensus reached | Yes | Yes | Low | Low |
| Amatya (2023) | CXR analysed by 2 radiologists + disagreements decided by a pediatric radiologist | Yes | Yes | Low | Low |

Flow and timing

|  | **Describe any patients who did not receive the index test(s) and/or reference standard or were excluded from the 2x2 table** | **Describe the time interval and any intervention between index test(s) and/or reference standard or who were excluded from the 2x2 table** | **Was there an appropriate interval between the index test and reference standard** | **Did all patients receive a reference standard?** | **Did all patients receive the same reference standard?** | **Were all patients included in the analysis?** | **Could the patient flow have introduced bias?** |
| --- | --- | --- | --- | --- | --- | --- | --- |
| Shah (2013) | 209 were enrolled, of which 2 refused participation and 7 were excluded (3 had incompete LUS, 1 left before CXR, 3 had loss of blinding) | Unclear | Unclear | Yes | No | Yes | Unclear |
| Guerra (2016) | Unclear | Unclear | Unclear | Yes | No | Yes | Unclear |
| Lissaman (2019) | 142 patients were identified. 38 of these were excluded (4 had prior chest X-ray, 34 did not give consent). Of the 104 patients enrolled, 7 were withdrawn (4 incomplete LUS, 3 consent was withdrawn) | LUS performed within 12 hours of the CXR | Yes | Yes | Yes | Yes | Low |
| Rodriguez-Contreras (2022) | Unclear | Within the same day | Yes | Yes | Yes | Unclear | Low |
| Samson (2018) | 34 excluded (no reason given) + 80 didn't get LUS for various reasons | Unclear | Unclear | Yes | Yes | Yes | Unclear |
| Amatya (2023) | 1 person excluded because CXR was uninterpretable | Unclear | Unclear | Yes | Yes | Yes | Unclear |

QUADAS 2- Risk of Bias

|  | **Risk of bias** | | | | **Applicability Concerns** | | |
| --- | --- | --- | --- | --- | --- | --- | --- |
|  | Patient selection | Index test | Reference standard | Flow and timing | Patient selection | Index test | Reference standard |
| Shah (2013) | Low | Unclear | Low | Unclear | Low | Low | Low |
| Guerra (2016) | Low | Unclear | Low | Unclear | Low | Unclear | Low |
| Lissaman (2019) | Unclear | Unclear | Low | Low | Low | Low | Low |
| Rodriguez-Contreras (2022) | Unclear | Low | Low | Low | High | Low | Low |
| Samson (2018) | Low | Unclear | Low | Unclear | Low | Low | Low |
| Amatya (2023) | Low | Unclear | Low | Unclear | Low | Low | Low |
